# Efficient suilysin-mediated invasion and apoptosis in porcine respiratory epithelial cells after streptococcal infection under air-liquid interface conditions

**DOI:** 10.1038/srep26748

**Published:** 2016-05-27

**Authors:** Fandan Meng, Nai-Huei Wu, Maren Seitz, Georg Herrler, Peter Valentin-Weigand

**Affiliations:** 1 Institute of Virology, University of Veterinary Medicine Hannover, Hannover, Germany; 2 Institute of Microbiology, University of Veterinary Medicine Hannover, Hannover, Germany

## Abstract

Streptococci may colonize the epithelium in the airways and other entry sites. While local infection often remains asymptomatic, severe or even fatal diseases occur when streptococci become invasive and spread to different sites in the infected host. We have established porcine respiratory air-liquid interface cultures (ALI) from the porcine lung to analyze the interaction of streptococci with their primary target cells. As representative of the streptococcal family we chose *Streptococcus suis* (*S. suis*) that is not only a major swine respiratory pathogen but can also infect humans. Suilysin, a cholesterol-dependent cytolysin (CDC), is an important virulence factor. By comparing a *S. suis* wt strain with a suilysin-deficient mutant, we demonstrate that suilysin contributes to (i) adherence to airway cells (ii) loss of ciliated cells (iii) apoptosis, and (iv) invasion. Furthermore, we show that cytolytic activity of suilysin is crucial for these effects. A striking result of our analysis was the high efficiency of *S. suis*-induced apoptosis and invasion upon infection under ALI conditions. These properties have been reported to be less efficient when analyzed with immortalized cells. We hypothesize that soluble effectors such as suilysin are present at higher concentrations in cells kept at ALI conditions and thus more effective. These results should be relevant also for infection of the respiratory tract by other respiratory pathogens.

By taking up air from the environment, the respiratory tract is regularly exposed to various microorganisms and thus has a potential risk to get infected by pathogenic bacteria or viruses^1^. Streptococci are important pathogens that may initiate respiratory tract infections^2^. They comprise a number of diverse members infecting humans and/or animals^3^,^4^,^5^. In general, they have evolved effective mechanisms to adhere to and colonize different mucosal surfaces^6^,^7^. Adherence to surface-exposed host structures often results only in asymptomatic infections^6^,^8^,^9^. Severe diseases occur when streptococci succeed to invade the deeper host tissues^10^,^11^. Disease symptoms may range from pharyngitis, scarlet fever, and toxic shock syndrome caused by *S. pyogenes*, also referred to as group A streptococci. Further manifestations are peumonia, meningitis, or sepsis caused by *S. agalactiae* (group B streptococci) and *S. pneumoniae* (pneumococci)^11^,^12^,^13^,^14^. Additionally, animal streptococci may have a zoonotic potential and induce disease not only in their host but also in humans. *Streptococcus suis* (*S. suis*) is a major cause of meningitis, sepsis, arthritis and pneumonia in piglets with a worldwide distribution^3^,^15^. Moreover, it can cause severe infections to people that are in close contact with infected pigs. Two outbreaks of severe human infections by *S. suis* have occurred in China and affected several hundred persons in 1998 and 2005. The mortality rate was 56% and 18.6%, respectively^10^,^16^. In Vietnam, *S. suis* infections have been reported to be the most common cause of bacterial meningitis in adults^17^. In Thailand, *S. suis* also is considered as an emerging human pathogen^18^. Most cases of animal and human *S. suis* infections were caused by serotype 2 strains and some cases have been attributed to serotype 14^18^,^19^,^20^. Nevertheless, it has been only poorly studied which virulence factors of *S. suis* contribute to the early stages of infection, i.e. adherence to and colonization of mucosal surfaces or to crossing the mucosal epithelia to generate systemic disease.

The portal of entry most often used by streptococci for invasion is the airway system. The lower respiratory tract is usually sterile because the mucociliary clearance system based on the concerted action of mucus secretion and ciliary activity provides an effective defense mechanism^6^. Although the respiratory epithelium is known to be the main target tissue of respiratory pathogens, the role of the well-differentiated airway epithelial cells has been poorly studied^21^. The well-differentiated airway epithelium is composed of different types of cells, such as mucus-producing (goblet) cells, basal cells, ciliated and non-ciliated cells. Primary culture systems for well-differentiated respiratory epithelial cells provide the closest *in vitro* representation of the airway epithelium and thus are the cell cultures of choice to analyze respiratory infections^21^,^22^,^23^,^24^.

Pathogenic streptococci are characterized by the release of cholesterol-dependent pore-forming cytolysins (CDC). Pneumolysin, the CDC of *Streptococcus pneumoniae* induces cytotoxic effects and apoptosis of macrophages and neuronal cells^25^. Furthermore, it may promote bacterial adherence and invasion^26^,^27^. Suilysin, the CDC of *S. suis*, plays a role in the pathogenesis of invasive disease caused by this bacterial agent^28^,^29^. It is responsible for cytotoxic effects and contributes to the adhesion of *S. suis*^30^,^31^,^32^,^33^,^34^. However, little is known about the role of suilysin in infection of the airway epithelium.

Studies addressing streptococcal invasion have been performed mainly with immortalized cell lines^31^,^35^,^36^. We have recently used precision-cut lung slices to analyze the adherence of *S. suis* to differentiated porcine airway epithelial cells^37^. To further elucidate the question how *S. suis* invades the respiratory tract, in the present study air-liquid interface (ALI) cultures were used. ALI cultures of human airway epithelial cells are well established for analysis of virus infections. Porcine ALI cultures have been used only recently for infection by influenza viruses and adenoviruses^23^,^38^. Compared to the human counterpart, the latter cells have the advantage that the source of cells is well-defined and reproducible as far as the age and genetic background of the animals are concerned.

Here we show that infection of differentiated airway epithelial cells by *S. suis* at ALI conditions results in suilysin-mediated effects, such as adherence, cytotoxicity, invasion, and apoptosis. Strikingly, some of the effects are very efficient when compared to those reported in studies with conventional culture systems.

## Results

### Polarization and differentiation of porcine airway epithelial cells *in vitro*

**Figure 1:**
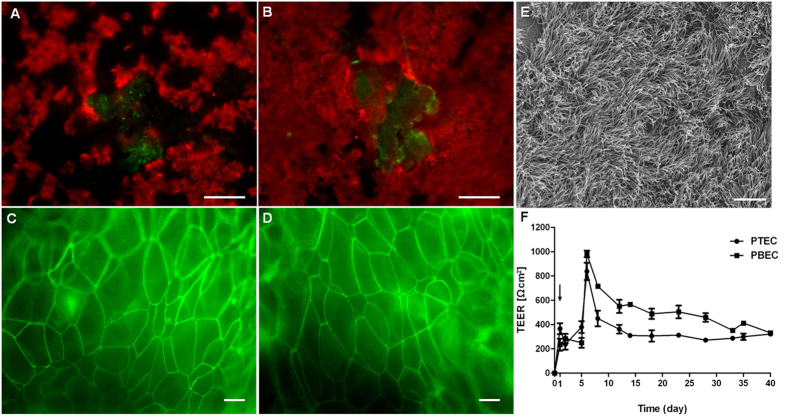
Immunofluorescence microscopy analysis and TEER measurement of well-differentiated porcine tracheal and bronchial epithelial cells (PTEC and PBEC) grown under air–liquid interface (ALI) conditions. Porcine epithelial cells from PTEC or PBEC, were grown to confluence on filter supports and maintained under ALI conditions for five (**A–D, F**) or seven weeks. The extent of differentiation was analyzed by visualizing cilia (**A,B,E**) and mucus (**A,B**), tight junctions (**C,D**), and by measuring the transepithelial resistance (**F**). For analysis by immunofluorescence microscopy, PTEC (**A,C**) and PBEC (**B,D**) were stained for cilia by anti-β-tubulin antibodies (**A,B**, red) and for mucus (**A,B**, green), respectively. Tight junctions were visualized by staining for occludin (**C,D**, green). (**E**) Analysis of PBEC by FESEM differentiated for 7 weeks. (**F**) TEER values of PTEC and PBEC during differentiation. The day of cell seeding is indicated by arrowhead (day 1). Bars represent 50 μm (**A,B**) and 10 μm (**C–E**).

To establish a culture system for differentiated porcine airway epithelial cells, primary porcine tracheal and bronchial epithelial cells (PTEC and PBEC, respectively) were cultured in a transwell filter system. When the cell monolayer had reached confluence, the medium was removed from the apical compartment and – under these ALI conditions - the cells started to differentiate. PTEC and PBEC were maintained at ALI conditions for at least 5 weeks. The presence of cilia, which is considered as an indicator of the extent of differentiation, was determined by immunofluorescence microscopy (Fig. 1A,B). Interestingly, cilia expression (in red) was more pronounced on PBEC (Fig. 1B) in comparison to PTEC (Fig. 1A). Immature short cilia were first observed on cells at the border of the filter supports of cultures maintained for 7 days under ALI conditions (data not shown). The amount of cilia further increased until week 7 of cultivation. During the differentiation process, the monolayer adopted the appearance of a pseudostratified epithelium. Mucin production (in green) was demonstrated by mucin-5 AC staining after 5 weeks of culture under ALI conditions in both PTEC (Fig. 1A) and PBEC (Fig. 1B). Moreover, the presence of tight junctions was demonstrated by a positive staining for occludin (Fig. 1C,D), reflecting morphological stability and barrier function of PTEC and PBEC. As shown in Fig. 1E, scanning electron microscopy (SEM) revealed mature cilia at a high percentage of the epithelial surface of well-differentiated PBEC 7 weeks after cultivation under ALI conditions. A peak value of transepithelial electrical resistance (TEER) at 6 days of culture under ALI conditions indicated that PTEC and PBEC had adopted a polarized organization (Fig. 1F). Afterwards TEER values decreased to some extent, but remained constant over the remaining observation period of 40 days. Taken together, we established well-differentiated porcine airway epithelial cells under ALI conditions *in vitro,* which closely resemble the cells of the airway epithelium.

### Colonization of well-differentiated PTEC and PBEC by *S. suis*

The infection of porcine well-differentiated airway epithelial cells by *S. suis* was first analyzed by determining bacterial adherence and colonization under ALI conditions. PTEC and PBEC were infected with encapsulated wt strain 10, its isogenic suilysin-deficient mutant strain 10Δsly and the nonencapsulated suilysin-deficient mutant 10ΔcpsΔsly. In addition we tested two complemented strains expressing suilysin with single amino-acid substitutions, one of which carried a silent mutation (suilysin derivative S148) and the other one which resulted in lack of cytolytic macropore formation (suilysin derivative W461F). The strains were generated by complementation of strain 10Δsly with either of two point-mutated suilysin genes, designated strain cS148, and strain cW461F, respectively. We included those strains to confirm possible effects of suilysin by complementation (strain cS148) and to elucidate whether effects might require cytolyctic activity of the toxin or not (strain cW416F). In previous studies we observed that suilysin derivative W461F does not induce any cytolytic pores^39^, and that suilysin derivative S148 is similarly active as the wt suilysin (unpublished results).

PTEC and PBEC were infected with *S. suis* strains from the apical side at an multiplicity of infection (MOI) of 20. After 4 hours, cells were washed thoroughly to remove non-adherent bacteria, and from then on, cells were kept under ALI conditions for at least 72 hours. As shown in Figure S1, at the indicated times colonizing bacteria on the apical side of cells were quantified by plating. The wt strain and all mutant strains were able to colonize the apical side of PBEC, since the bacterial numbers remained constant over the whole observation period of 72 hours. Similar results were obtained with PTEC (results not shown). No differences in bacterial growth were observed between the parental strain and the different mutants incubated with either of the two cell types (shown for PBEC in Figure S1). These results showed that *S. suis* can efficiently colonize the apical side of PTEC and PBEC under ALI conditions with similar kinetics. Our findings indicate that this culture system for well-differentiated airway epithelial cell can be applied to analyze *S. suis* infection *in vitro*.

### Different sensitivity of PTEC and PBEC to the cytotoxic effect induced by suilysin of *S. suis*

**Figure 2:**
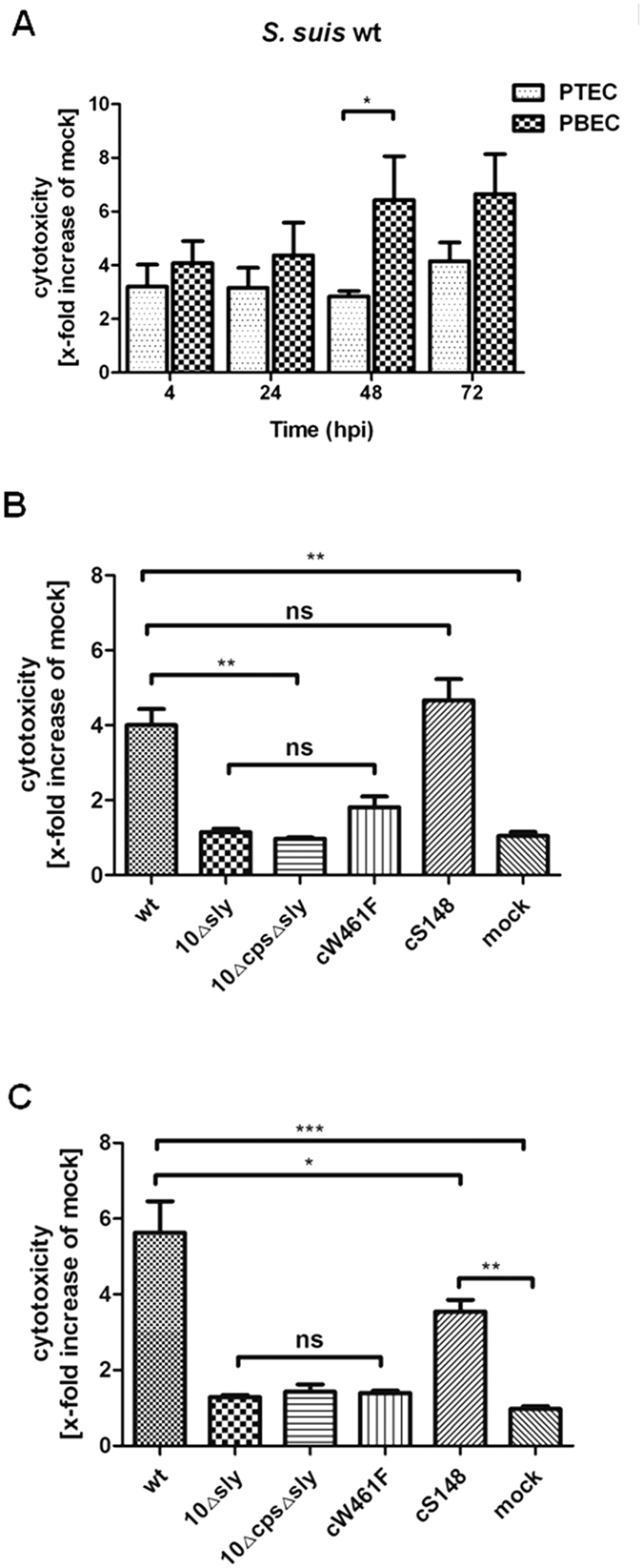
Cytotoxic effects of *S. suis* on well-differentiated porcinebronchial epithelial cells. Cytotoxic effects of streptococci on PBEC were quantified by a standard LDH-release assay. (**A**) Time-dependent cytotoxic effects of *S. suis* wt on PTEC and PBEC. Cytotoxic effects of *S. suis* wt, 10Δsly,10ΔcpsΔsly, cW461F and cS148 on PBEC, respectively, at 4 hpi (**B**) and at 48 hpi (**C**). Results are expressed as x-fold increase compared to mock-infected cells (mock). Results are expressed as means ± SEM and significance, indicated by *(P-value < 0.05), **(P-value < 0.01), ***(P-value < 0.0001), was determined using one-way-ANOVA and Tukey multiple comparison test. Experiments were performed at least three times.

Cytotoxicity was examined by determining the release of cellular lactate dehydrogenase (LDH), a widely used marker for cellular damage. PTEC and PBEC were infected with *S. suis* wt, either of the mutants (10Δsly and 10ΔcpsΔsly), or either of the complemented mutant strains cW461F and cS148, respectively, and analyzed for cytotoxic effects in comparison to mock-infected cells. As shown in Fig. 2A, cytotoxicity induced by the wt strain increased in a time-dependent manner from 4 hpi to 72 hpi. LDH release induced by the wt strain at 4 hpi in PBEC (4-fold increase) was similar to that in PTEC (3-fold), but at 48 hpi it was significantly higher in PBEC (7.3 fold) than in PTEC (2.3 fold). To elucidate whether cytotoxic effects would also influence integrity of the cellular barrier, we determined the TEER of PTEC and PBEC, which is indicative of the formation of tight junctions and an intact epithelial barrier^23^. Results revealed that mock-infected PTEC and PBEC remained unaffected until end of the experiment (96 hpi). In comparison, TEER of PTEC and PBEC infected with the wt strain showed a significant decrease starting at 72 hpi and 48 hpi, respectively, whereas that of PTEC/PBEC infected by either of the two suilysin-deficient mutants was similar to the control (Figure S2).

Results of LDH release and TEER determinations suggest that PBEC are more susceptible to cytotoxic effects of *S. suis*. Therefore, in the following experiments we focused on the effects of *S. suis* on PBEC. As expected, the two suilysin-deficient mutants 10Δsly, 10ΔcpsΔsly had no detectable cytotoxic effect on PBEC, even at 48 hpi (Fig. 2B,C). Notably, of the complemented mutant strains expressing point-mutated suilysin cW461F and cS148, respectively, the former showed no cytotoxic effects, whereas the latter induced a significant LDH release at both 4 and 48 hpi (Fig. 2B,C). Thus, cytotoxic effects on differentiated airway epithelial cells were dependent on the presence of suilysin and required cytolyctic activity, i.e. macropore-formation, of the toxin.

### Adherence of *S. suis* to well-differentiated porcine bronchial epithelial cells

**Figure 3:**
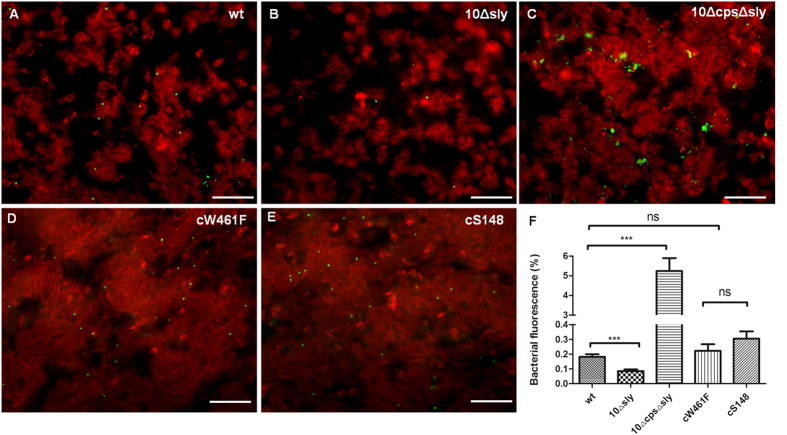
Adherence of *S. suis* to well-differentiated porcine bronchial epithelial cells. PBEC were apically infected with approximately 1 × 10^7 ^CFU of *S. suis* wt, 10Δsly, 10ΔcpsΔsly, cW461F or cS148 respectively, After 4 h, cells were washed thoroughly to remove non-adherent bacteria. To determine adherence of streptococci to PBEC (**A–E**), immunostaining was applied to visualize cilia (red) and streptococci (green). Bars represent 50 μm. To Quantify the bacteria attached to PBEC (**F**) the areas containing green-fluorescent bacteria were determined from panels (**A**). Results are expressed as means ± SEM and significance, indicated by ***(P < 0.0001), was determined using one-way-ANOVA and Tukey multiple comparison test. Experiments were performed at least three times.

Adherence to host cells is important for *S. suis* to cause infection and initiate disease^40^,^41^. The ability of *S. suis* to adhere to and colonize well-differentiated epithelial cells was determined by immunofluorescence microscopy at 4 hpi. PBEC were apically infected with 1 × 10^7 ^CFU of *S. suis* per filter. After incubation at 37 °C for 4 h, cells were washed thoroughly to remove non-adherent bacteria and fixed with 3.7% formaldehyde. As shown in Fig. 3A, immunofluorescence analysis demonstrated that streptococci were able to adhere to PBEC. Bound bacteria were mainly detected on ciliated cells. As shown in Fig. 3F, quantification of adherent bacteria revealed that suilysin-positive *S. suis* wt strain adhered to PBEC (0.16%) with significantly higher efficiency than the suilysin-deficient mutant 10Δsly (0.075%), respectively. The number of adherent wt bacteria was 2-fold higher than that of 10Δsly on PBEC cells. This result is consistent with a previous report that suilysin promotes the adherence of *S. suis* to the immortalized respiratory epithelial cell line HEp-2 and porcine *ex vivo* precision-cut lung slices^37^,^39^. Interestingly, adherence of the two complemented strains cW461F and cS148, respectively, was similar to that of the wt and significantly higher than that of the suilysin-deficient mutant 10Δsly. These results suggest that macropore-formation of suilysin was not involved in promotion of *S. suis* adherence to PBEC. On the other hand, it has been reported that nonencapsulated *S. suis* strain showed higher capacity of adherence to and invasion of host cells in comparison to the parental strain^42^,^43^. Therefore, we also included a suilysin-deficient nonencapsulated mutant (10ΔcpsΔsly). As shown in Fig. 3A, lack of a capsule enhanced the adherence of the suilysin-deficient mutant to PBEC. Quantification of microscopic examinations confirmed significantly higher adherence of the nonencapsulated mutant 10ΔcpsΔsly as compared to the encapsulated wt and the mutant 10Δsly (Fig. 3F). These results indicate that suilysin and the capsule can both affect the adherence of *S. suis* to well-differentiated epithelial cells. Suilysin shows an enhancing effect on *S. suis* adherence to airway epithelial cells, which is independent of macropore-formation, whereas the capsule has a reducing effect.

### Polar invasion of differentiated airway epithelial cells by *S. suis* causes cell damage and impaired barrier function

**Figure 4:**
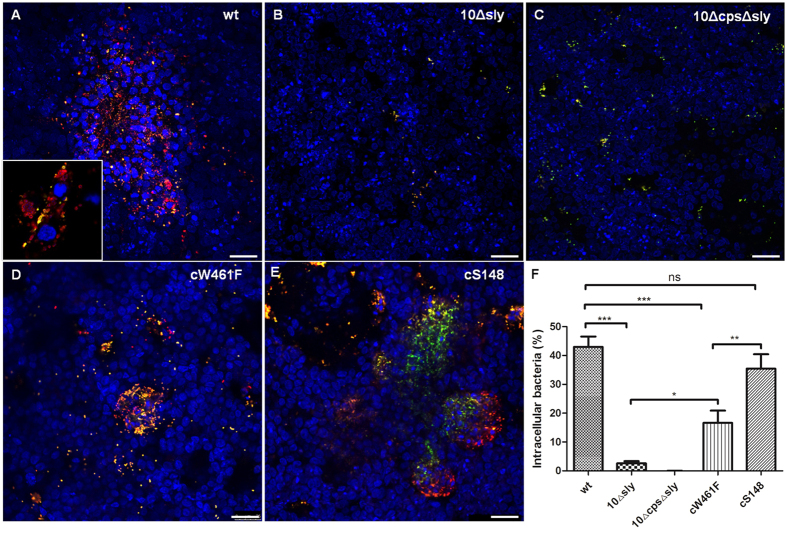
Polar invasion of *S. suis* of well-differentiated porcine airway epithelial cells. PBEC were apically infected with approximately 1 × 10^7 ^CFU of *S. suis* wt, 10Δsly, 10ΔcpsΔsly, cW461F or cS148 respectively. After 4 h, cells were washed thoroughly to remove non-adherent bacteria, and then further incubated until 48 hpi under air-liquid interface conditions. The infection of PBEC (**A–E**) were immunostained for streptococci both before permeabilization to visualize adherent bacteria (yellow/green) and after permeabilization to visualize invasive bacteria (red). Nuclei were stained by DAPI (blue). Bars represent 25 μm. To quantify invasive *S. suis* in PBEC (**F**), the percentage of intracellular bacteria was determined by comparing the red fluorescent bacterial signals from panels A to the total bacteria number. Results are expressed as means ± SEM and significance, indicated by ***(P < 0.0001), was determined using one-way-ANOVA and Tukey multiple comparison test. Experiments were performed at least three times.

The site of entry most often used by streptococci for invasion is the airway system. Thus, invasion of PBEC by streptococci was analyzed by double immunofluorescence microscopy. As shown in Fig. 4A,D,E, intracellular bacteria (stained red) were detected in PBEC after infection with suilysin-positive *S. suis* wt and the two complemented strains cW461F and cS148, respectively.

Interestingly, both suilysin-negative mutants, 10Δsly and 10ΔcpsΔsly, were hardly found intracellularly (Fig. 4B,C). Quantification of the microscopic data revealed that more than 40%, 18% and 35% of all detected bacteria were located intracellularly in PBEC in the case of the suilysin-positive wt strain, strain cW461F and strain cS148, respectively (Fig. 4F). In contrast, invasion rates of both suilysin-deficient mutants were significantly lower (2.6% for 10Δsly, below detection limit for 10ΔcpsΔsly). The clear difference between wt and mutant *S. suis* was also found in the invasion of PTEC, for which the rate of intracellular wt bacteria was 28% (data not shown). We conclude that suilysin is crucial for efficient invasion of *S. suis* into airway epithelial cells, and that cytolytic activity of suilysin contributes significantly to this effect, since strain cW461F was significantly less invasive than wt strain and strain cS148. Nevertheless, there was a significant difference between invasion rates of both suilysin-negative mutants and strain cW461F (Fig. 4F), suggesting that there may be invasion-promoting effects of suilysin which do not depend on cytolytic activity.

**Figure 5:**
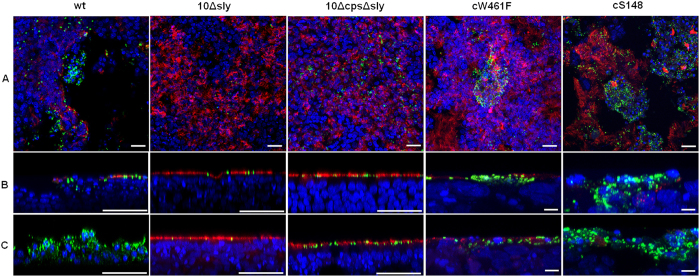
Suilysin-dependent damage of PBEC after infection with *S. suis*. PBEC were apically infected with approximately 1 × 10^7 ^CFU of *S. suis* wt, 10Δsly, 10ΔcpsΔsly, cW461F or cS148 respectively. After 4 h, cells were washed thoroughly to remove non-adherent bacteria, and further incubated under air-liquid interface conditions until 48 hpi (**A,B**) and 96 hpi (**C**). For analysis by confocal laser scanning microscopy, immunostaining was performed to visualize cilia (red) and streptococci (green). Nuclei were stained by DAPI (blue). Bars represent 25 μm in horizontal sections (**A**) and 50 μm (larger bar) or 5 μm (short bar) in vertical sections (**B,C**). Experiments were performed at least three times.

To further dissect to what extent suilysin-induced cytotoxic effects may contribute to deeper invasion of *S. suis*, we co-stained streptococci and ciliated cells of PBEC at 48 hpi and 96hpi. In these experiments the suilysin-positive wt strain and the complemented strain cS148 induced damage to the PBEC resulting in the loss of a large proportion of ciliated cells at 48hpi (Fig. 5A). Vertical sections of these samples illustrate that the loss of the ciliated cells enables the wt and cS148 bacteria to invade to deeper areas of the cell layer (Fig. 5B). Strain cW461F was able to invade PBEC to a certain extent and caused only moderate local lesions at 48 hpi. In striking contrast, the suilysin-deficient mutants 10Δsly and 10ΔcpsΔsly remained adherent to ciliated cells and were unable to cross this barrier. This effect was more pronounced at 96 hpi (Fig. 5C), when just a few cilia were detectable and invasive *S. suis* wt even reached the basal area of the epithelial cell layer. Similar results were obtained with strain cS148, i. e. many bacteria crossed the epithelial barrier. Accordingly, despite large numbers of adherent bacteria at 96 hpi, the suilysin-deficient nonencapsulated mutant 10ΔcpsΔsly was unable to invade the ciliated epithelium. Interestingly, infection of PBEC by the point-mutated suilysin cW461F for 96h revealed many intracellular bacteria but hardly any damaged ciliated epithelial cells or bacteria invading deeper areas (Fig. 5). These results indicate that subcytolytic effects of suilysin contribute to the invasion of *S. suis* into PBEC. However, macropore-formation seems to further promote tissue invasion by causing epithelium damage.

### Suilysin-dependent induction of apoptosis in differentiated airway epithelial cells by *S. suis* infection

**Figure 6:**
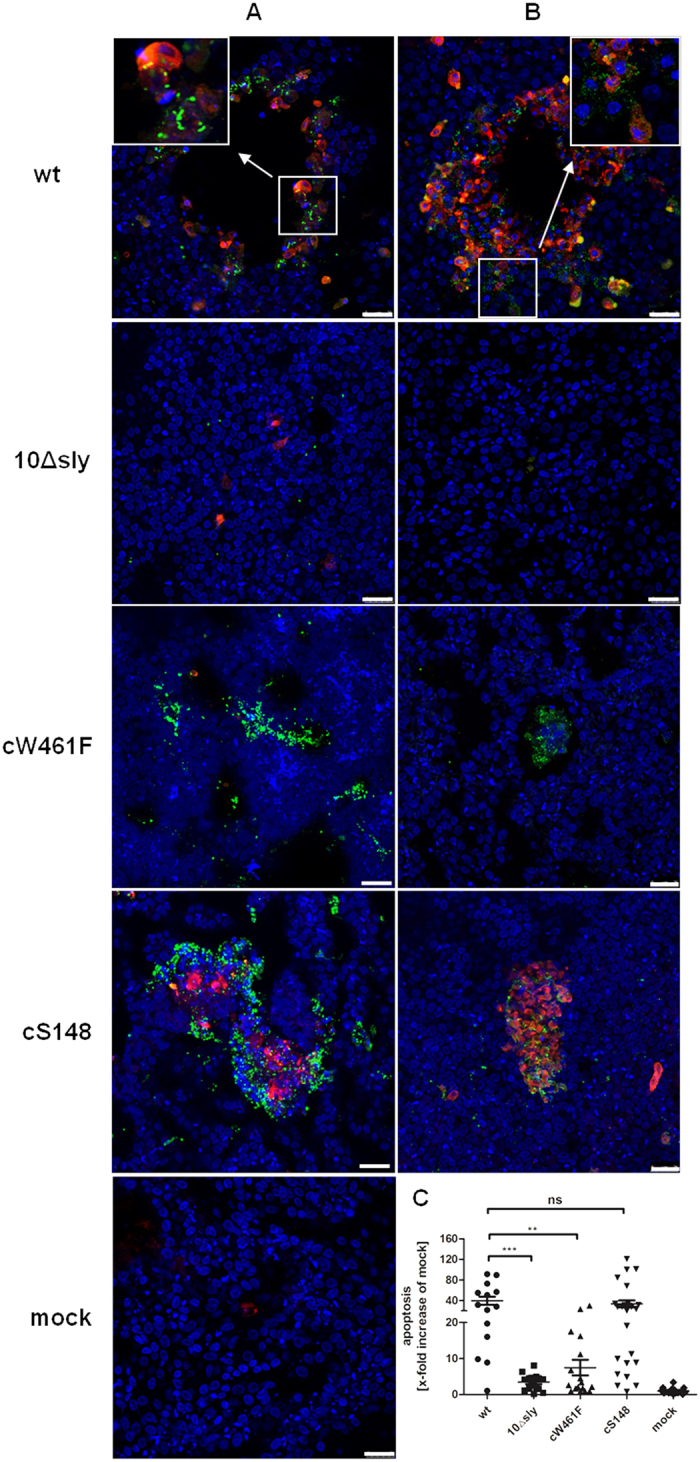
Detection of apoptosis in *S. suis* infected porcine bronchial epithelial cells. PBEC were apically infected with approximately 1 × 10^7^ CFU of *S. suis* wt, 10Δsly, cW461F or cS148 respectively. After 4 h, cells were washed thoroughly to remove non-adherent bacteria, and further incubated until 48 hpi under air-liquid interface conditions. Colocalization of *S. suis* and apoptotic cells in PBEC **(A).** Analysis by confocal laser scanning microscopy, cells were stained for cleaved caspase-3 (red) and streptococci (green). Nuclei were stained by DAPI (blue). Bars represent 25 μm. Colocalization of suilysin and apoptotic cells of PBEC (**B).** For analysis by confocal laser scanning microscopy, cells were immunostained for cleaved caspase-3 (red) and suilysin (green). Nuclei were stained by DAPI (blue). Bars represent 25 μm. Quantification of apoptotic cells in PBEC **(C).** To quantify the apoptotic cells on PBEC, the areas containing red-fluorescent cells were determined from panels A and B. Results are expressed as x-fold increase compared to mock-infected cells (mock). Results are expressed as means ± SEM and significance, indicated by ***(P < 0.0001), was determined using one-way-ANOVA and Tukey multiple comparison test. Experiments were performed at least three times.

To get more detailed information about the mechanism of suilysin-induced cellular damage processes, we analyzed whether infection of PBEC by *S. suis* results in apoptosis. For this, we studied the presence of the cleaved form of caspase-3 in PBEC, which is considered as a major enzyme regulating apoptosis^44^. As shown in Fig. 6, the number of cells positive for cleaved caspase-3 (in red) were most abundant in PBEC infected by wt and cS148 *S. suis*. Most of the stained cells were located at the edge of areas where the epithelial cell layer had been damaged and cells had detached as a consequence of infection. Cells positive for capase-3 showed further signs of apoptosis such as chromatin condensation and apoptotic bodies. Furthermore, the wt strain and strain cS148 clearly colocalized with caspase-3 positive cells of PBEC. In contrast, only few cells were stained for cleaved caspase-3 in PBEC infected with the suilysin-deficient mutant 10Δsly and strain cW461F, no colocalization of caspase-3 with bacteria was observed (Fig. 6A).

To find out whether suilysin was involved in the apoptosis induced by *S. suis*, we analyzed colocalization of cleaved caspase-3 with suilysin by confocal microscopy. As shown in Fig. 6B, in wt and complemented strain cS148 infected PBEC, suilysin (in green) was detected by immunostaining and colocalized with cells that had been also stained for cleaved caspase-3 (in red). High density of membrane-associated suilysin was detectable on apoptotic cells, which is consistent with the localization of suilysin-positive wt streptococci in invasion areas described above (Fig. 6A). As expected, no suilysin and only few cleaved caspase-3 positive cells were detected in PBEC infected by the suilysin-deficient mutant 10Δsly. Interestingly, accumulated membrane-associated suilysin was detected in the complemented strain cW461F infected PBEC, but this accumulated suilysin did not induce increased apoptotic cells (as shown in Fig. 6C). Quantification of apoptotic cells revealed that suilysin-positive *S. suis* wt strain and complemented strain cS148 induced a 40-fold and 35-fold increase of apoptotic cells compared to mock infection of PBEC, respectively. Increases were significantly higher than those caused by the suilysin-deficient mutant 10Δsly (5-fold) and complemented strain cW461F (8-fold). There was no difference in increase of apoptotic cells between the latter two strains. These results indicate that suilysin induces apoptosis in PBEC and suggest that this effect requires macropore-formation activity of suilysin.

## Discussion

Aim of this study was to establish the ALI culture system as an infection model for analyzing bacterial infection of porcine well-differentiated respiratory epithelial cells. ALI cultures from porcine airway cells have only recently been described for infection studies with viruses such as influenza virus and adenovirus^23^,^38^ but not yet for bacterial infections. *S. suis* had been analyzed using a variety of immortalized respiratory epithelial cells^12^,^31^,^32^,^39^,^42^, as well as polar porcine choroid plexus epithelial cells and human choroid plexus papilloma cells^45^,^46^. Recently, we used precision-cut lung slices (PCLS) as a culture system to analyze the co-infection of porcine differentiated respiratory epithelial cells by swine influenza virus and *S. suis*^37^. PCLS are maintained in culture medium, whereas ALI cultures acquire their nutrients from the medium only via the basolateral membrane, and the apical side of the cells has contact to air. In this respect the ALI system most closely resembles the situation of the airway epithelial cells in their respective host. We found that streptococci efficiently grow on the apical side of the airway epithelial cells in the absence of apical culture medium. Therefore, ALI cultures are an appropriate model system to analyze the infection of well-differentiated airway epithelial cells by *S. suis*. In the future, it should be interesting also to study the infection by other bacterial pathogens.

Adherence to and colonization of host cells is important for *S. suis* to cause infection and initiate disease^40^,^41^. Although the respiratory epithelium is the main target tissue of respiratory pathogens, the role of the well-differentiated airway epithelial cells for bacterial infection has been poorly studied^21^. Here we show that suilysin promotes the adherence to and invasion of ALI cultures of porcine airway epithelial cells by encapsulated and nonencapsulated *S. suis*, which is consistent with a previous study using immortalized cells^39^. Ciliated cells were found to be the primary target cells for *S. suis* adherence.

A striking result was the high capacity of *S. suis* to invade differentiated airway epithelial cells, as reflected by invasion rates ranging between 28% and 40% determined for PTEC and PBEC, respectively. This result was unexpected since our previous study on host cell invasion of *S. suis* using the human cell line HEp2 revealed a rather low invasive capacity^39^. However, we cannot directly compare invasiveness of *S. suis* in both culture systems, since quantification was done by different methods due to the models applied in the present and the previous study. Because of the very low invasion of immortalized cells, encapsulated *S. suis* is considered as an extracellular bacterium^31^,^39^,^42^. It has been suggested that invasion by this streptococcus requires the down-regulation of the capsule to achieve efficient adherence and subsequent invasion. Following invasion, the capsule would have to be upregulated again to ensure bacterial survival in the blood stream. However, this has never been demonstrated *in vivo*. Our data show that encapsulated *S. suis* is very efficient in invasion suggesting that differential expression of the capsule may not be essential for efficient invasion.

The invasion of epithelial cells by streptococci involves bacterial uptake by a dynamic process of membrane reorganization, possibly related to the formation of membrane ruffles that have been observed in *S. suis* infected HEp-2 cells^39^. This uptake mechanism is suilysin-dependent. As suilysin is a soluble protein, the high efficiency of this process in the case of ALI cultures can be explained by the higher local concentration of this protein in the absence of apical medium. In the case of immortalized cells, suilysin produced by *S. suis* is immediately diluted by the medium and, thus, a homogeneous and low concentration of the protein is available for interaction with the cell surface. By contrast, suilyin released from *S. suis* to the apical side of airway epithelial cells in ALI cultures will not be diluted by the culture medium and, thus, can accumulate and be active on the site where it is released from the bacteria.

Apart from its role in adherence and invasion, suilysin is also responsible for the cytotoxicity of *S. suis*. CDC are important components of pathogenic streptococci. The soluble monomeric forms assemble to ring-shaped oligomeric structures on the surface of the cell^47^. Upon insertion of the oligomers into the plasma membrane, the resulting pore formation perforates the membrane and exerts a cytotoxic effect. In the case of suilysin, cytotoxicity has been demonstrated with different types of immortalized cells^30^,^31^,^32^,^33^. Our results demonstrate a pronounced cytotoxic effect also for well-differentiated airway epithelial cells. Notably, this effect was absent in a strain expressing a derivative of suilysin which carries a point-mutation (W461F) of the domain responsible for macropore-formation. In fact, immunostaining revealed large amounts of cell surface-located suilysin molecules, at least some of which might represent formation oligomeric pore complexes. The large amount of suilysin may again be explained by the ALI culture conditions that enable high local concentrations of the toxin. The strong cytotoxic effect of suilysin also explains the lesions that were observed at 24 hpi in the aiway epithelial cell layer that expanded until the whole epithelium was damaged. Future studies have to analyze why the cellular damage appeared to be more pronounced in bronchial cells than in tracheal cells.

The damage of the epithelial cells promotes streptococcal infection in different ways. It results in the loss of their barrier function and allows the bacteria to get access to deeper areas of the epithelium that are usually not exposed to the environment. This conclusion is supported by our infection studies with PCLS, another culture system for differentiated airway epithelial cells^37^. It has been suggested that dead cells may provide a rich source of nutrition and growth factors and thus enhance streptococcal growth^48^. We found larger numbers of bacteria in areas where the epithelium had been damaged. Interestingly, the infection with the complemented strain cW461F, which could only express subcytolytic toxin effects, revealed a high number of invasive bacteria in PBEC but only moderate damaging effects. These results demonstrate that macropore-formation acticity and subcytolytic effects of suilysin promote invasion of *S. suis.*

Streptococcal cytolysins are known to induce apoptotic cell death. In the case of *S. suis,* reports about apoptotic effects are very limited^49^,^50^. Apoptosis may be triggered by a variety of physiological and pathological stimuli^44^,^51^. Our results show that infection by *S. suis* induces suilysin-dependent apoptosis which is very likely to be responsible for the damage and death of differentiated epithelial cells. Results of experiments with the complemented strain cW461F revealed that macropore-formation of suilysin is required for induction of apoptosis in airway epithelial cells. However, at present it remains unclear what the underlying mechanisms are. In *S. pneumoniae* a pneumolysin-induced calcium influx has been shown to mediate apoptosis in neuronal tissues^52^,^53^. Thus, it is plausible to speculate that suilysin-induced apoptosis in airway epithelial cells may be initiated by calcium influx through the pores in the plasma membrane. Calcium influx induced by suilysin was observed in HEp-2 cells (unpublished data). Whether this can be translated to PBEC remains to be clarified in future studies.

Taken together, our results show that suilysin affects the infection of well-differentiated respiratory epithelial cells by *S. suis* in different ways. Comparing our present results with those of previous studies using conventional cell culture systems, it seems that the efficiency of the suilysin-mediated effects are greatly enhanced when cells are maintained under ALI conditions. These findings have important implications for understanding the pathogenesis of streptococci and may stimulate efforts to analyze the infection of highly differentiated airway epithelial cells by other bacterial agents.

## Material and Methods

If not stated otherwise all materials were purchased from Sigma-Aldrich.

### Bacterial strains

The virulent *S. suis* serotype 2 wild-type strain 10 (designated as wt) was kindly provided by H. Smith, Lelystad, NL^54^. The corresponding suilysin-deficient mutant of wt strain 10 (designated as 10Δsly) and suilysin-deficient unencapsulated mutant of wt strain 10 (designated as 10ΔcpsΔsly) were constructed as described in previous studies^35^,^39^. In all infection experiments, cryo-conserved bacterial stocks were used and prepared as previously reported^37^. Streptococci were grown on Columbia agar supplemented with 7% sheep blood (Oxoid).

*S. suis* strain 10cS148Δsly (designated cS148) and 10cW461FΔsly (designated cW461F) were constructed by chromosomal complementation of the *S. suis* suilysin-deficient mutant 10Δsly by allelic exchange. The amplicon slyBamHI_SphI_cS148 was generated using oligonucleotide primers PM_sly_SphIfor (CCCGCATGCATGAGAAAAAGTTCGCACTT) and PM_sly_BamHIrev (CCCGGATCCTTACTCTATCACCTCATCCG). The amplicon slyBamHI_SphI_cW461Fmax was generated with oligonucleotide primers sly_pSET5s_SphIfor (CCCGCATGCAGTATAATACATTGCCAGAT) and sly_pSET5s_BamHIrev (CCCGGATCCTATTCCGGAAATTGTTGGAA). DNA of *S. suis* wt 10 was used as template. The amplicons were cut with the restriction enzymes indicated in the name of the primers and inserted into the corresponding sites of pSET5s vector^55^. Oligonucleotide primers SLYcompSMfor (ATTCTCCAACAAGATCAACTGTTCGAACAGG) and SLYcompSMrev (CCTGTTCGAACAGTTGATCTTGTTGGAGAAT) encoding the silent mutation S148 in the suilysin gene and oligonucleotide primers slyTrp-Phefornew (TACAGGATTAGCATTTGAGTGGTGGAGAAC) and slyTrp-Pherevnew (GTTCTCCACCACTCAAATGCTAATCCTGTAC) encoding the W461 to F461 substitution were used to generate point-mutations by site-directed-mutagenesis according to the instruction manual of QuikChange® Site-Directed Mutagenesis Kit (Stratagene, Ja Jolla, CA). Restriction analysis and sequencing was performed with pSET5s_sly_S148 and pSET5s_sly_W461F, respectively, to verify both constructs. The allelic exchanges for generation of 10cS148Δsly (cS148) and 10cW461FΔsly (cW461F) were performed essentially as described previously^56^. Final sequencing was performed to verify the resulting *S. suis* strains cS148 and cW461F, respectively.

### Porcine airway epithelial cell culture

Primary porcine PTEC or PBEC were isolated from the trachea and bronchi of pigs, respectively. Porcine lungs were obtained from a local slaughterhouse at Hannover. The animals had a good health status and no clinical symptoms. Tissue segments were washed with PBS and digested in Eagle’s minimal essential medium (EMEM) supplemented with 100 units/ml penicillin and 100 μg/ml streptomycin, amphotericin B (50 μg/ml), gentamicin (50 μg/ml), 100 μg/ml DNase (Roche) and 1 mg/ml Protease for 48 hours at 4 °C^23^. Primary PTEC or PBEC were harvested by scraping the cells from trachea or bronchi with scalpel, respectively. Cells were propagated in collagen I pre-coated flasks until they reached confluence with Bronchial Epithelial Cell Growth Medium (BEGM), as previously described^57^,^58^ with some modifications, which is Bronchial Epithelial Cell basal Medium (BEBM, Lonza) supplemented with the required additives (Sigma). BEGM was refreshed at every 2 days intervals. Approximately after 4 days of cultivation cell monolayers reached 80% confluence and were subjected for further differentiation. Primary PTEC or PBEC were dissociated with trypsin (Life Technologies) and resuspended with Air–Liquid Interface medium (ALI medium), a mixture 1:1 of BEGM and Dulbecco’s modified Eagle’s medium (DMEM, Life Technologies) and 2.5 × 10^5 ^cells were seeded on type IV collagen pre-coated 0.33 cm^2^ polyester transwell filters with polycarbonate membrane (0.4 μm pore size, Corning Costar). Cells were then maintained at 37 °C in a humidified atmosphere of 5% CO_2_. After the cells reached confluence, ALI medium was removed from the apical compartment and cells were cultured under the ALI conditions, the PTEC or PBEC were cultured at least for additional 5 weeks for differentiation at 37 °C in a humidified atmosphere of 5% CO_2_. ALI medium in the basolateral compartment was renewed every 2 or 3 days and the apical surface was washed once per week with Hanks’ balanced salt solution (HBSS, Life Technologies).

### Measurement of TEER

Confluent epithelial cells at ALI conditions developed a TEER, indicating the development of tight junctions and an intact epithelial barrier^23^. The TEER of well-differentiated PTEC and PBEC, respectively, was measured before and after infection with *S. suis* by using the Millicell ERS-2 (Electrical resistance system, Millipore). For TEER measurement, both the apical and basolateral compartment of the transwell system were washed 3 times with PBS+. Then 200 μl ALI medium was added to the apical compartment while 500 μl were added to the basolateral compartment. TEER across the multilayer of uninfected PTEC and PBEC was monitored from the first day after seeding cells on transwell filters over 40 days to characterize polarization and differentiation of PTEC and PBEC, respectively. After infection of cells with *S. suis* TEER was measured for at least 96 hpi.

### Growth of *S. suis* on well-differentiated epithelial cells

For each treatment three transwell filters were used and all experiments were repeated three times. Well differentiated porcine airway epithelial cells (at least 5 weeks of differentiation) were maintained without antibiotic and antimycotics one day before infection and washed three times with phosphate-buffered saline supplemented with calcium (PBS+) before inoculation with *S. suis*. Well-differentiated epithelial cells were inoculated from the apical side of the filter with approximately 1 × 10^7 ^CFU of *S. suis* strain wt, 10Δsly, 10ΔcpsΔsly, cW461F, cS148 in 70 μl ALI medium MOI of approximately 20 bacteria per epithelial cell), meanwhile, cells inoculated with ALI medium only served as mock infected cells. Infection was performed for 4 hours in humidified atmosphere containing 5% CO_2_ at 37 °C. Afterwards, infected and mock infected cells were washed three times with PBS+ to remove non-adhered bacteria. All cells were maintained under ALI one space in between conditions for up to 72 hours in humidified atmosphere containing 5% CO_2_ at 37 °C.

To determine bacterial replication and growth kinetics, supernatants from the apical compartment of infected transwell filters were collected before the washing step (4 hpi) and plated on Columbia agar supplemented with 7% sheep blood. At the later time points (24, 48, and 72 hpi) 70 μl PBS+ was added to the apical filter compartment and cells were incubated on a horizontal shaker for 5 min to collect non-adherent streptococci. Supernatants were plated as described above.

### Cytotoxicity assay

Cytotoxicity was detected by an LDH-release assay as described previously^39^. Supernatants from the apical compartments of infected and mock infected transwell filter were collected before the washing step at 4 hpi. For the determination of cytotoxicity at 24, 48, 72 hpi 70 μl PBS+ were added to the apical filter compartment and cells were incubated on a horizontal shaker for 5 min to collect the supernatant. The LDH release was determined using the Cytotox^®^ 96 assay kit (Promega). The experiments were performed in triplicates and repeated at least three times. To quantify the relative cellular damage of well-differentiated epithelial cells, results were related to uninfected control samples and expressed as x-fold increase of mock infected samples.

### Immunofluorescence microscopy and field emission scanning electron microscopy (FESEM)

The following primary antibodies were used in this study: Cy3-labeled monoclonal antibody against β-tubulin (1:400), mucin-5 AC antibody (1:250, Santa Cruz Biotechnology), polyclonal antibody against cleaved caspase-3 (1:400, Cell Signaling), occludin alexa fluor 488 conjugate antibody (1:200, invitrogen), polyclonal rabbit serum raised against suilysin (1:1300)^35^, and a polyclonal rabbit serum raised against S*. suis* (1:200)^35^. As secondary antibodies green fluorescent anti-rabbit IgG secondary antibody (Alexa Fluor^®^ 488 anti-rabbit IgG (H+L) antibody (1: 1000, Life Technologies) and red fluorescent anti-rabbit IgG secondary antibody (Alexa Fluor^®^ 568 anti-rabbit IgG (H+L) antibody (1:1000, Life Technologies) were used.

Double immunofluorescent microscopy (DIF) of *S. suis* infected PTEC and PBEC was performed as previously described^42^ with some modifications. All incubation steps were performed at RT. All Infected cells were washed three times with PBS+ and fixed with 3.7% formaldehyde in PBS for 20 min. Formaldehyde-fixed preparations were washed and 0.1 M glycine in PBS was added for 5 min, after then samples were washed 3 times with PBS and followed by incubation in blocking buffer containing 1% bovine serum albumin (BSA) in PBS to block nonspecific binding sites. All antibodies were diluted in 1% BSA in PBS. Blocking buffer was removed and preparations were incubated with rabbit anti-*S. suis* antiserum diluted 1:200 for 1h. After washed with PBS, samples were incubated for 45 min with green fluorescent anti-rabbit IgG secondary antibody Alexa Fluor^®^ 488 anti-rabbit IgG (H+L) antibody 1:1000 diluted (Life Technologies) to stain extracellular streptococci. Following, the preparations were washed with PBS and permeabilized with 0.2% Triton X-100 for 20 min. Then preparations were again incubated with the rabbit anti-*S. suis* antiserum 1:200 diluted for 1h, washed with PBS, then incubated with red fluorescent anti-rabbit IgG secondary antibody (Alexa Fluor^®^ 568 anti-rabbit IgG (H+L) antibody 1:1000 diluted (Life Technologies)) for 45 min to stain intra- and extracellular bacteria. After PBS washing steps, the preparations were incubated with DAPI (4′,6-diamidino-2-phenylindole) and after final washing, the membrane of transwell filters were cut down and embedded in Mowiol and stored at 4 °C for further analysis.

Samples were analyzed by using the inverse immunofluorescent microscope Nikon Eclipse Ti-S equipped with a 10x/0.30 and 40x/0.60 Plan Fluor objectives (Nikon). Subsequently, the area of the epithelial cells surface positive for green fluorescent bacteria was analyzed by applying the analySIS^®^ 3.2 software (Soft Imaging System) to quantify bacterial adherence. Three areas were chosen randomly for each sample and all experiments were repeated three times. Confocal immunofluorescence microscopy of samples were performed using a TCS SP5 confocal laser scanning microscope equipped with a 63x/1.30 NA glycerin HC PL APO objective and a 63x/1.40 oil HCX PL APO objective (Leica). Image stacks with a z or y-distance of 0.5 μm per plane were acquired using 1 Airy unit pinhole diameter in sequential imaging mode to avoid bleed through. Maximum intensity projections were calculated for display purposes and adjusted for brightness and contrast using ImageJ/Fiji.

FESEM studies of uninfected PBEC was performed as described previously^42^.

### Statistical analyses

If not stated otherwise, experiments were performed at least three times and results were expressed as means with standard deviations. Data were analyzed by one-way-ANOVA and Tukey multiple comparison test, using the GraphPad Prism 5 software. A p-value < 0.05 was considered significant.

## Additional Information

**How to cite this article**: Meng, F. *et al*. Efficient suilysin-mediated invasion and apoptosis in porcine respiratory epithelial cells after streptococcal infection under air-liquid interface conditions. *Sci. Rep.* **6**, 26748; doi: 10.1038/srep26748 (2016).

## Supplementary Material

Supplementary Information
