# Supplementary material for: Efficient suilysin-mediated invasion and apoptosis in porcine respiratory epithelial cells after streptococcal infection under air-liquid interface conditions
Source: Sci Rep. 2016 May 27;6:26748. doi: 10.1038/srep26748 (PMC4882623; doi:10.1038/srep26748)
Supplement: Supplementary Information [file srep26748-s1.pdf]

Efficient suilysin-mediated invasion and apoptosis in porcine respiratory epithelial cells after streptococcal infection under air-liquid interface conditions

Fandan Meng, Nai-Huei Wu, Maren Seitz, Georg Herrler, Peter Valentin-Weigand

- Supplementary Information -

Figure S1

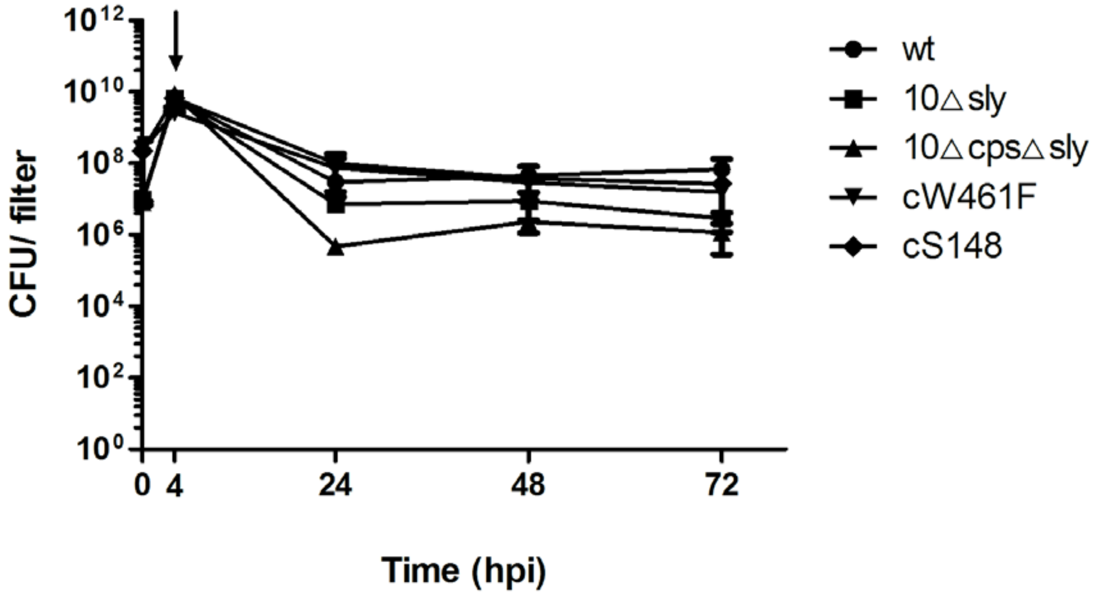

Colonization of *S. suis* on the apical side of well-differentiated porcine bronchial epithelial cells.

Growth kinetics of streptococci determined by counting of colony forming units (CFU) after replica plating on blood agar plates on the apical side of infected PBEC maintained under air-liquid interface conditions. PBEC were apically infected with approximately 1 x 10^7 CFU of the *S. suis* wt strain, 10Δsly, 10ΔcpsΔsly, cW461F or

cS148 respectively, and cells were washed thoroughly after 4 hours (indicate by arrowhead) to remove non-adherent bacteria. Cells were further incubated until 72 hpi under air-liquid interface conditions.

Figure S2

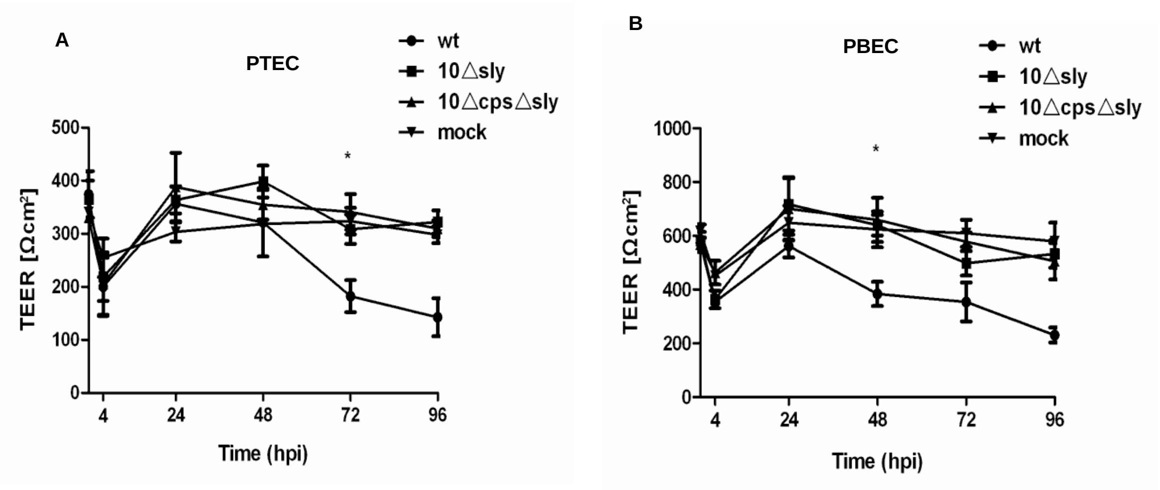

TEER measurement of *S. suis*-infected differentiated porcine airway epithelial cells (PTEC and PBEC)

PTEC and PBEC were apically infected with approximately 1 x 10^7 CFU of *S. suis* wt strain, 10Δsly, or 10ΔcpsΔsly, respectively. After 4 h, cells were washed thoroughly to remove non-adherent bacteria, and further incubated until 96 hpi under air-liquid interface conditions. TEER of mock-infected and *S. suis*-infected PTEC (A) or PBEC (B) was determined at the indicated time points. Results are expressed as means ± SEM and significance indicated by * (P<0.05) was determined using one-way-ANOVA and Tukey multiple comparison test.
